# Efficient discovery of responses of proteins to compounds using active learning

**DOI:** 10.1186/1471-2105-15-143

**Published:** 2014-05-16

**Authors:** Joshua D Kangas, Armaghan W Naik, Robert F Murphy

**Affiliations:** 1 Lane Center for Computational Biology, Carnegie Mellon University, 5000 Forbes Ave., Pittsburgh, PA 15213, USA; 2 Departments of Biological Sciences, Machine Learning and Biomedical Engineering, Carnegie Mellon University, Pittsburgh, PA, USA; 3 Freiburg Institute for Advanced Studies and Faculty of Biology, Albert Ludwig University, Freiburg, Germany

**Keywords:** Active learning, Machine learning, Drug development, Polypharmacology, Computational biology, Drug discovery

## Abstract

**Background:**

Drug discovery and development has been aided by high throughput screening methods that detect compound effects on a single target. However, when using focused initial screening, undesirable secondary effects are often detected late in the development process after significant investment has been made. An alternative approach would be to screen against undesired effects early in the process, but the number of possible secondary targets makes this prohibitively expensive.

**Results:**

This paper describes methods for making this global approach practical by constructing predictive models for many target responses to many compounds and using them to guide experimentation. We demonstrate for the first time that by jointly modeling targets and compounds using descriptive features and using active machine learning methods, accurate models can be built by doing only a small fraction of possible experiments. The methods were evaluated by computational experiments using a dataset of 177 assays and 20,000 compounds constructed from the PubChem database.

**Conclusions:**

An average of nearly 60% of all hits in the dataset were found after exploring only 3% of the experimental space which suggests that active learning can be used to enable more complete characterization of compound effects than otherwise affordable. The methods described are also likely to find widespread application outside drug discovery, such as for characterizing the effects of a large number of compounds or inhibitory RNAs on a large number of cell or tissue phenotypes.

## Background

Drug discovery and development is a lengthy process that begins with the identification of potential drug targets and ends after testing in clinical trials. The targets are generally identified through basic science studies as being critical components affected in a disease. Once a target protein has been identified, the goal is to identify drug-like compounds that either increase or decrease its activity. High throughput screening (HTS) and high content screening (HCS) are frequently used to ascertain the effects of many compounds on a target. However, even with automation, screening a large experimental space can be expensive (especially for HCS). One approach to reducing the need for experimentation is to generate a model for compound effects *in silico*, a process referred to as virtual screening. There are two common methods [1]. During a quantitative structure activity relationship (QSAR) analysis, molecules are checked for the presence or absence of specific structural elements. The vector describing a molecule is referred to as a “fingerprint.” QSAR methods have been used to make predictions about the activity of compounds on target proteins [2,3]. Molecular docking is an alternative method that requires knowledge of the structure of both target and compound [4,5]. Computer simulations are run in which the target and compound are forced into contact and the interaction energy between the target and compound molecule estimated. These methods take into consideration features of the target protein and potential drugs. Beyond virtual screening, efforts have also been made to apply machine learning techniques to the wealth of information available in the PubChem database, paying particular attention to the gross imbalance of active to inactive compounds [6,7] in efforts to make accurate predictions of the effects of compounds on targets.

These predictive studies consider the effects of many compounds on one (or a small number) of targets in order to identify promising compounds for further development. However, it is not uncommon in drug development for previously unknown effects to be discovered after significant investment in a potential drug, resulting in relatively high attrition rates in later phases or even after drug release [8]. These side effects are not discovered earlier because screening is for *desired* effects of compounds on a single target protein without considering whether compounds have *undesired* effects on other targets. This suggests that early drug screening should consider a larger portion of the compound-target effect space [9]. Ideally, we would have knowledge of the whole experimental space of compounds and targets (which we can represent as a matrix with rows for each target (~10^4^) and columns for each compound (~10^6^)).

By having knowledge of all effects of all compounds, much more informed decisions could be made about which compounds to advance through the development process (including the possibility that a desired drug should have *more than one* effect as well as minimal side effects). However, measuring the full matrix would require on the order of 10^10^ measurements, the cost of which would be prohibitive. An alternative is clearly needed.

As with single targets, predictive modeling methods for the larger space have also been described. Chemogenomic approaches have been developed that concurrently consider the similarity of compounds and the similarity of ligands to make predictions for unknown associations between proteins and compounds [10]. Furthermore, methods have been developed that allow for the identification of compounds with a desired effect profile across multiple targets by using evolutionary methods to generate compounds to be tested [11]. These polypharmacological methods make predictions for the effects of compounds across multiple targets. Using text mining methods, clinical outcome records have also been analyzed to predict effects [12]. Inverse docking methods have been developed as well which start with a compound and measure the interaction energy between the compound and multiple proteins [13].

Building any of these predictive models requires data for at least a subset of all possible experiments. This is typically all data currently available, or new results for a human-specified subset thought to be representative. In approaches like those mentioned above, machine learning methods are then used to predict results for a large set of compounds, and a small number of these are tested. In most cases, the process stops after this, and selected compounds are advanced to further development. However, the process can be made iterative, so that information from the additional experiments may be used to improve the model, make new predictions and select more experiments to execute [14]. This type of approach is referred to as *active* learning in the machine learning literature. In active learning, rather than being chosen in advance, experiments are iteratively selected to most improve the accuracy of the predictive model. In the context of drug development, this should require fewer experiments to make accurate predictions (of both desired and undesired effects) allowing for more effective decisions and reduced late-phase attrition [9]. While active learning is widely used in some fields, there have been only limited applications to biological problems [15-21].

**Figure 1:**
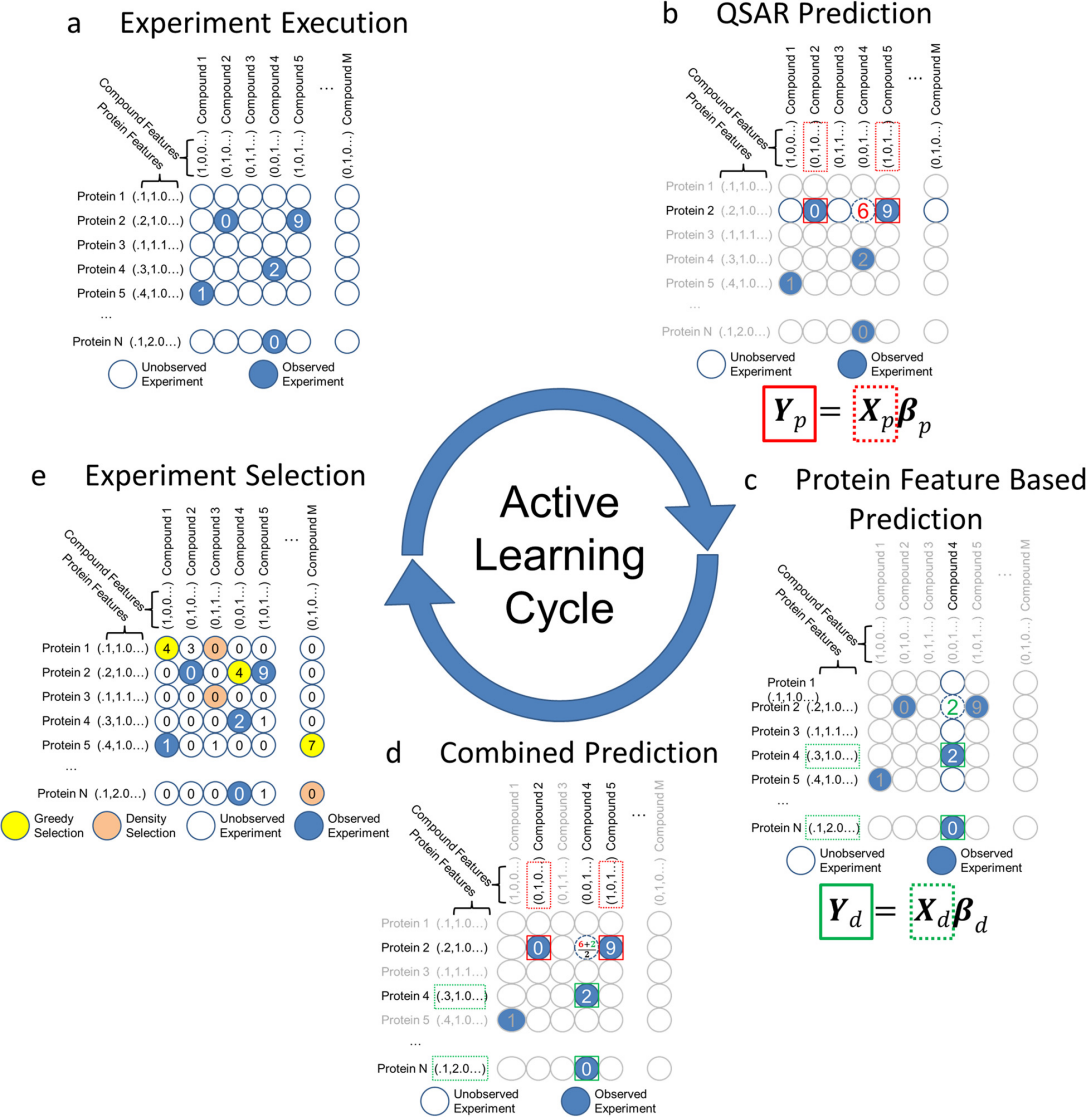
**An active learning pipeline for an experimental space with N proteins and M compounds. (a)** A round of active learning begins with the data for all of the experiments that have been observed so far. **(b)** A separate model is constructed for each protein using the compound features to make predictions for the effect of each compound on the activity of that protein. This is illustrated for Protein 2 for which regression using the observed experiments for Compounds 2 and 5 predicts that Compound 4 would show an activity of 6. This model is referred to as CFO. **(c)** A separate model is constructed for each compound using the protein features to make predictions for the effect of that compound on the activity of each protein. This is illustrated for Compound 4 for which regression using the observed experiments for Proteins 4 and N predicts that Protein 2 would show an activity of 2. This model is referred to as PFO. **(d)** For the CCT approach, if predictions from both methods are available, they are averaged. (In the early rounds when no experiments may have been observed for a given protein or compound, predictions from both models may not be possible). **(e)** The complete set of observations and predictions is shown, and experiments that would be chosen for the next round of acquisition by different methods are shown (greedy selection would pick the experiments with the highest predicted values, while density selection would pick experiments for compounds and proteins that are most different from those previously selected). The results for the chosen experiments will be added to those observed so far to begin the next round of active learning.

Active learning consists of three phases performed in a loop (as illustrated for the work described here in Figure 1). A campaign of experiments can be initialized either using prior results from literature or databases or by randomly selecting a batch of experiments from an experimental space. **(1)** A model is generated to represent the currently available data. **(2)** From that model, experiments are selected for execution that are expected to improve the model. **(3)** The set of experiments is executed and the resulting data are combined with previously collected experimental data. The loop then continues from Step 1 until either a desired accuracy of predictions is achieved or a specified budget has been exhausted. There have been limited previous applications of active learning to the drug discovery process. In these efforts, compound activity was considered to be binary (active or inactive) and effort was focused on only a single target [22,23].

The most important difference of the work described here from previous approaches is our emphasis on *active* machine learning to *simultaneously* model the effects of many compounds on many targets. To demonstrate the utility of active learning for drug discovery in the context of multi-target modeling, we combined two modeling approaches to make predictions about activities for large numbers of combinations of compounds and targets. Our model uses features developed for virtual screening to describe compounds, and features from sequence analysis to describe target proteins. As a part of this effort, we did not endeavor to make the most accurate predictive model possible. Rather, we investigated the utility of applying active learning in combination with predictive models in order to efficiently discover active compound-target pairs. In tests using data from the PubChem database, we found that active compound-target pairs could be discovered as much as twenty-four times faster using active learning than by random selection of experiments. The algorithms we describe are also computationally efficient, making application to very large experimental spaces practical.

## Results

### Dataset

To evaluate our proposed approaches, we chose to use existing experimental results for assays on many targets and many compounds. We therefore began by assembling a large set of compound effect scores from PubChem (http://pubchem.ncbi.nlm.nih.gov). In total, compound activity scores for 177 assays were assembled. Of these assays, 108 were from *in vitro* assays and 69 were from *in vivo* assays. Of the 600,000 compounds in PubChem across the 177 assays, an average of 30% had a reported activity score for a given assay. (We do not know but assume that the missing values are approximately missing at random.) Of these, we created a dataset of all assay data for 20,000 randomly-chosen compounds, resulting in a system with 3.5 million possible experiments (the distribution of scores across all compounds and assays is shown in Additional file 1). All combinations of target and compound with scores above 80 or below −80 were marked as hits. (Note that each PubChem assay includes its own rank score cutoff above which a chemical is considered to be “active”. Our cutoff of 80 is more stringent than that used for most assays.) Information on the assays, compounds and their respective features, can be found in the Additional files 2 and 3.

### Model definition

As an initial approach to constructing a predictive model, we explored using linear combinations of features. Given the large numbers of features involved, lasso regression [24] was used because it allows for efficient feature selection for linear regression models. We note that while the assay scores may be non-linearly related to true activity, and while estimates of true activity may be obtained by further manipulation or testing, we expect them to be good approximate predictors of which combinations of compounds and targets will show high activity.

Three approaches to prediction of the assay scores were used. The first approach used compound features only (CFO) to predict the activity of each compound in a given assay (analogously to QSAR). Using lasso regression, compound features were selected that were strongly indicative of the activity of a compound on a *single* target. A regression model was learned for each individual target allowing for the selection of compound features unique to a target (Figure 1b and Equation 1). The second approach used protein features only (PFO) to predict the effect on each target of a given compound. When considering all experiments which involved a single compound, lasso regression allowed us to select features of the target protein which were indicative of the likelihood for a target to be affected by that *single* compound (Figure 1c and Equation 2). The third approach made a combined compound-target (CCT) prediction by averaging the two predictions for each compound-target combination (Figure 1d and Equation 3).

### Evaluating model performance

We first sought to determine how accurately these models could predict target-compound hits as a function of how much training data was available. To do this, we randomly sampled a sequence of experiments in batches of 384 experiments until 3% of the experimental space had been sampled (note that each combination of assay and compound was considered independently when selecting random experiments). As each experiment was sampled, we combined it with all previous experiments from that sequence to train a model and evaluated its ability to predict hits for all remaining data.

**Figure 2:**
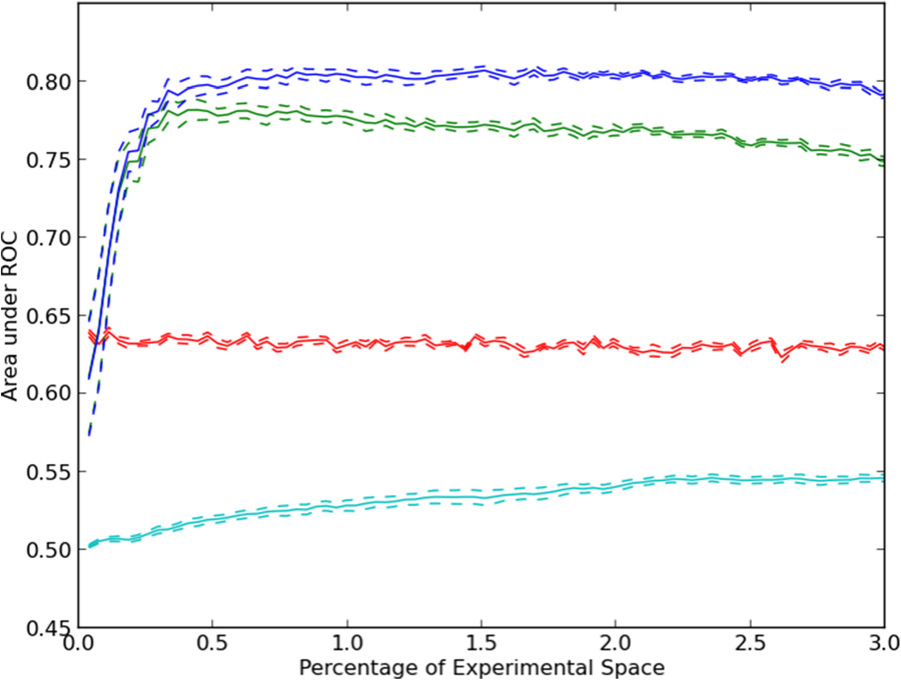
**Evaluation of prediction methods with increasing amounts of randomly selected training data.** Ten random sequences of experiments were used to select data used for training regression models. After each experiment was chosen, a ROC curve was constructed by gradually raising the threshold on the predicted assay score at which an experiment was considered to be positive. The mean and standard error of the area under the ROC curve for prediction of positive experiments after each experiment is plotted for each regression method. The prediction methods shown are: within target random prediction in red, regression using protein features only (PFO, cyan), regression using compound features only (CFO, blue) and CCT (green).

A receiver-operator characteristic (ROC) curve was calculated for each of these models by varying the classification threshold to predict a hit (note that only the prediction threshold was varied; the definition of an actual hit as having an absolute value above 80 was unchanged). Finally, the area under the ROC curve was calculated for each set of predictions. This process was repeated ten times for each of the three prediction approaches described above (CFO, PFO and CCT). The means and standard errors of the area under the ROC curve for the ten trials for each prediction approach are shown in Figure 2. Two methods can be considered to generate random predictions for comparison to these results. The simplest would be to randomly choose predictions from the set of all scores for all assays. Because these are globally random, the area under the ROC curve is clearly expected to be 0.5. All of our methods perform better than this. A more demanding baseline was therefore used, in which scores were randomly chosen from those for all compounds for a given target. The predictions from this sort of random predictor are expected to be more accurate than randomizing across all observations (since targets with a lot of hits will be randomly predicted to have a lot of hits), thus it is a more stringent standard for comparison. Predictions using CFO or using CCT performed better than random by this standard (Figure 2). This is despite the fact that less than 0.1% of the combinations were active according to our definition.

We also considered which features were more informative than others. To make a single set of predictions across the entire space of 20,000 compounds and 177 targets requires the training of 20,177 lasso regression models. The final models trained at 3% of the experimental space (from Figure 2) were analyzed and the proportion of models where the coefficient for each feature was non-zero was calculated. To determine the magnitude of the effect of a feature on prediction, the mean absolute coefficient for each feature (only when it was selected) was calculated. For targets, the most frequently selected features (and those with the largest coefficients) were the amino acid compositions. For compounds, the most frequently selected feature was “Group IIa (Alkaline earth)” and the feature with the largest absolute coefficient was “4 M Ring”. Further details on other features are provided in the Additional file 3.

**Figure 3:**
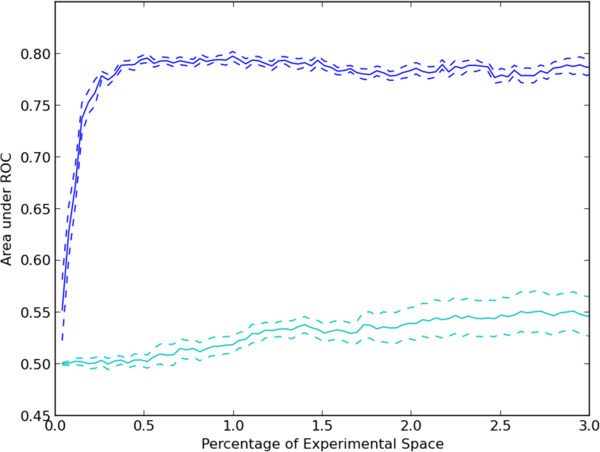
**Evaluation of prediction methods for held out targets or compounds with increasing amounts of randomly selected training data.** Ten random sequences of experiments were used to select data from compounds or targets not held out and used for training regression models. After experiments were chosen, a ROC curve was constructed by gradually raising the threshold on the predicted assay score at which an experiment was considered to be positive. The mean and standard error of the area under the ROC curve for prediction of positive experiments from held out targets or compounds are plotted for held out compounds (blue) or targets (cyan).

We also were interested in how applicable a trained model would be to a new target or a new compound. We utilized the same random sampling approach described above. However, for each of the ten trials, the experimental results were held out for a unique 10% of all targets or compounds and a ROC curve was calculated for only the held out experiments. The result of this process is that when targets are held out entirely, only PFO models can be used and likewise when compounds are held out entirely, only CFO models can be used. The results (Figure 3) show that when holding out entire compounds, relatively accurate predictions can be made about activities from the remainder. As expected from the results in Figure 2, the predicted activities for held out targets are much less accurate. Both, however, perform better than random prediction (AUC = 0.5). The results confirm that the regression approach can capture important information about compound effects, even when no information about a compound is provided during training. The fact that scores could be predicted better for new compounds than for new targets may be due to the fact that data was available for many more compounds than targets (and thus there is a higher chance that the model has already seen a similar compound than that it has seen a similar target).

### Active learning simulation

Given that our modeling approach performed better than random at predicting relative activity scores, we next determined whether it could be used to successfully drive an active learning process (i.e., to find hits faster than expected at random or maximize predictive accuracy rapidly). For this, simulations were run for an experimental space of all 177 assays (129 unique protein targets) and all 20,000 compounds. For this experimental space, rank scores from actual experiments executed were available in PubChem for 1,043,300 experiments out of 3,540,000 possible experiments. Experiments selected during simulations were restricted to those for which results were available; requests from an active learner for other experiments were skipped.

To initialize a simulation, all experimental results were hidden from the active learner as if they had never been executed. A set of 384 experiments were selected randomly for “execution.” During the execution phase (Figure 1a), results from selected experiments were “revealed” and used for training of a predictive model (Figure 1b-d). A new batch of experiments was then selected using one of a number of active learning methods (illustrated in Figure 1e and described in Methods). Finally, the data for the selected experiments were added to the pool of previously selected data and the loop continued until 3% of the possible experimental space was explored. Each round consisted of the selection of 384 experiments. Ten separate simulations were run for each experiment selection method, each starting out with a different set of initial experiments. At each round, the discoveries (combinations whose absolute activity score was greater than or equal to 80) were counted, and the mean count and associated standard error recorded as a function of the fraction of experimental space so far explored.

**Figure 4:**
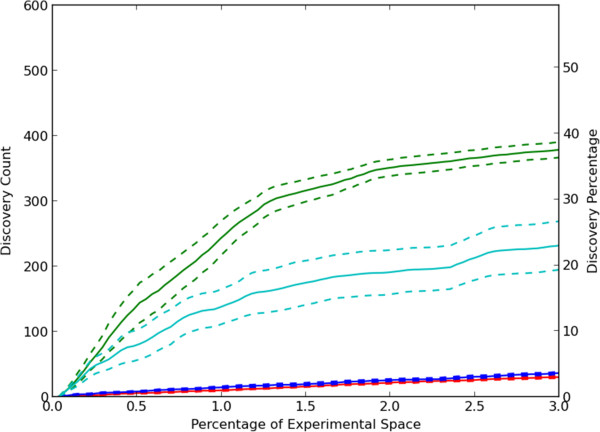
**Active learning to discover compound-target hits.** Experiments were selected which had the largest absolute predictions. The average number of discoveries and standard error for 10 separate trials are shown. The selection methods were random selection (red), CCT with greedy selection (green), greedy selection with single regression using only compound features for prediction (CFO, cyan) and greedy selection with single regression using only target features for prediction (PFO, blue).

We first considered a greedy active learning approach in which unobserved experiments that had the greatest predicted effect (inhibition *or* activation) were selected for measurement in the next round. This greedy approach was used in combination with CCT, single regression with predictions from compound features for each protein target (CFO) and single regression with predictions from protein target features for each compound (PFO). For comparison, a random selection method was also included. As shown in Figure 4, the greedy CCT method performed better than the other methods using less sophisticated predictive models. After exploration of 3% of the experimental space, an average of approximately 38% of possible discoveries were made. Results for the single regression approaches are also shown. As might be expected from the results in Figure 2, results for prediction from target features only are nearly the same as for random selection. Results using CFO are much better, but CCT performs even better. This may be considered surprising given that CFO performed better than CCT when measuring the accuracy of predictions based on the area under the ROC curve in Figure 2. However, the primary reason is that at high thresholds, predictions using CCT had a higher true positive rate than those of CFO giving the results in Figure 4. For the entire set of predictions across many thresholds, CFO predictions performed better as shown in Figure 2.

The rate of discovery for the greedy method using CCT decreased as the simulations progressed. Exploration of the experimental space with the greedy algorithm was limited to regions of the feature space which were predicted to have large activities. We considered the possibility that this limited the system’s ability to learn a better model, and that this could be overcome by acquiring data in regions where few observations have been made or where the model predictions were uncertain. Therefore, a “density-based” approach was also tested which selected experiments so as to explore the experimental space efficiently without regard to predicted values or experimental results. In this approach experiments were tested which were most similar to the unobserved experiments and least similar to observed experiments [25]. A variation on this idea, diversity sampling, was also tested, along with uncertainty sampling in which experiments with the highest uncertainty of their prediction were selected. Results for these approaches are shown in Additional file 4. The uncertainty-based selection method performed much better than random but not as well as CCT with greedy sampling. Density-based and diversity-based sampling performed similarly to random selection. These three classical active learning methods are generally designed to select experiments for execution which will yield the most accurate model, while the results in Additional file 4 are for finding hits. We therefore considered the accuracies of the models for each method by calculating the area under the ROC curve (as previously described for Figure 2). As shown in Additional file 5, all selection methods, except for uncertainty sampling, resulted in an initial peak accuracy followed by a slight, gradual reduction in the accuracy of the models. The better performance of uncertainty sampling compared to CCT with greedy sampling is consistent with the opposite result in Additional file 4. This is because uncertainty sampling does not prefer finding hits over non-hits.

**Figure 5:**
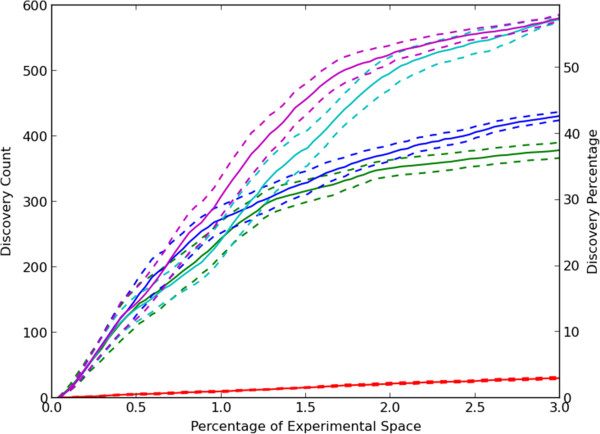
**Discovery rates for improved active learning approaches.** The average number of discoveries and standard error for 10 separate trials are shown. The methods were random choice (red), greedy CCT (green), greedy CCT-uncertainty hybrid (blue), and greedy selection-uncertainty hybrid using memory limits of five (cyan) and ten rounds (magenta).

Because uncertainty, diversity and density-based selection methods were designed to select experiments which would yield a more accurate predictive model, we also tested hybrids of greedy CCT with each of these methods. These hybrid methods were designed to concurrently improve the predictive model and confirm predictions generated by the increasingly accurate predictive model. The hybrids with density and diversity performed worse than greedy CCT by itself (Additional file 6) but the hybrid with uncertainty sampling performed slightly better (Figure 5).

**Figure 6:**
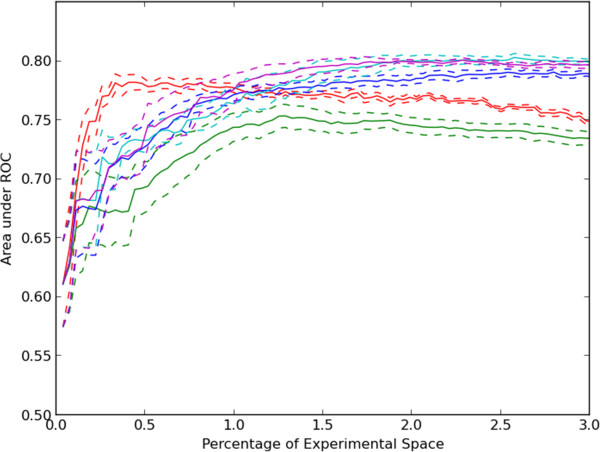
**Accuracy of models constructed using sampling by active learning approaches.** A CCT model was trained using all experiments chosen by each selection method up to a given round. The mean and standard error of the area under a ROC curve for each selection method is plotted versus the fraction of experiments performed up to that round. The methods were random choice (red), greedy CCT (green), greedy CCT-uncertainty hybrid (blue), and greedy selection-uncertainty hybrid using memory limits of five (cyan) and ten rounds (magenta).

We also considered the possibility that the decrease in rate of learning for greedy CCT was due to excessive testing of a given target for new discoveries after all of them have already been revealed. To address this possibility, we developed a modified approach (which we termed “limited memory”) in which only information from a given number of previous rounds was used in the model generation and active learning process. Any requests from the active learner for experiments previously selected and subsequently hidden were skipped. As shown in Figure 5, limiting memory to only the previous 5 or 10 rounds yields significant improvement in the discovery rate. Almost 60% of discoveries were made after only 3% of the experimental space was explored. We also found that limiting memory in the context of hybrid uncertainty methods also improved the quality of the predictive model as measured by the area under the ROC curve in Figure 6.

For reasons of computational time, we restricted our analysis to 20,000 compounds. It was therefore of interest to estimate how performance might change if more compounds were included. As a preliminary indication of this, we performed simulations for *smaller* sets of compounds. The results (Additional file 7) show that the learning rate is significantly worse for 5,000 compounds than for 20,000, but that it is not much different for 10,000 than 20,000. This suggests performance for larger sets might be as good or better.

## Discussion and conclusions

We have described a pipeline for executing experiments driven by an active learning system and demonstrated that it can result in the rapid discovery of compounds which affect target proteins using a set of heterogeneous assays. We found that the selection of experiments based only on predictions calculated using compound features (predicting the effect of a compound on a single target) performed significantly better than the selection of experiments based only on predictions from target features (predicting the sensitivity of a target protein to a single compound). Decent performance of the prediction models using compound features is to be expected given past results with QSAR approaches to modeling compound activity on a given target. The comparatively poor performance of the protein models could be a result of multiple issues: poor features, limited data, and heterogeneous data sources. The system included only features that could be calculated from sequence information, and it is likely that this feature set could be improved by the inclusion of features calculated from protein structural information. Some assays utilized in this study included high content screening assays in which living cells were imaged to measure the effects of compounds. These types of experiments are inherently more complex than simple binding assays and may have been poorly represented by features only describing a single target protein within the complex system. Both types of models performed better than random prediction and combining them yielded accurate models that could be utilized to rapidly make new discoveries. Previously, ensembles of predictors have yielded good results and the performance of these combined models may be caused by the same effect. Importantly, the addition of memory limitations to these models further improves the discovery rate. In this experiment, only information from 177 assays was used. As information from more assays becomes available, predictive models are expected to improve.

There are at least five factors to be considered in applying active learning approaches to problems such as compound screening. First, whether to use *a priori* measures of similarity between compounds or targets must be decided. The advantage of using them is that predictions can be made even before any data are acquired, but the disadvantage is that they may be biased towards previously explored compounds or targets. In separate work, we have described approaches for using modeling and active learning without such features [26]. Second, the method for choosing experiments to perform should reflect the goals of the campaign. As we have illustrated here, uncertainty sampling can be used to learn an accurate predictive model very efficiently. However, when the goal is not to learn an accurate predictive model of the whole space, but rather something such as just finding hits, we have also illustrated how hybrid experimentation selection methods can prove very beneficial. With hybrid methods, a portion of the experiments are chosen so as to learn an accurate predictive model and the remainder of the experiments are chosen to take advantage of the improved predictive model to accomplish the desired goal. Further, we have shown that limiting the memory of the active learning system can result in further improvements in efficiency by avoiding exploration of areas of the experimental space in which most relevant information has already been discovered. Third, computational complexity is an important consideration in practical use of active learning methods. Methods that model the entire space at once are theoretically preferable [27,28], but they can require prohibitively extensive computation for problems with thousands of targets and millions of compounds. In such cases, the methods we have described here can provide a faster alternative. Fourth, the logistics of the types of experimentation to be undertaken need to be considered. For example, in this study with the batch size we chose, 3% of the experimental space would have required approximately 80 rounds of experimentation. For some types of experimentation, a large number of small rounds may not be practical and thus larger batch sizes could be used in fewer rounds.

Finally, the primary goal of the active learning process is to reduce the experimentation required to complete an objective. In order for those reductions to be realized one needs to determine when to stop running experiments. This is an ongoing area of study, but progress has been made in our prior work [26]. In the current study, we observed that the discovery was high as the first 1.5% of the experimental space was explored and then decreased (but still occurred at a substantial rate). To explore whether the learning would continue or would plateau, we continued the simulations past 3% for the best method (greedy-uncertainty hybrid with 10 round memory limit). The learning rate continued at a rate about 2–3 times as fast as for random sampling and did not reach a plateau (data not shown). Extrapolating the learning rate predicts that it would find all hits after sampling approximately 20% of the experimental space.

It is worth noting that while simultaneous consideration of multiple targets and multiple compounds may *increase* the number of experiments needed to find a compound that affects a single target, it may be expected to *decrease* the average number of experiments per target when used to simultaneously conduct campaigns for multiple targets.

The selection of an appropriate batch size is an important consideration for the utilization of an active learning system. If there is a significant setup cost for a set of experiments (as is typically the case for HTS and HCS), then larger batches are preferable. If on the other hand, setup costs are low and a short time is required to execute the experiments relative to computational time to update the model, a smaller batch size would be preferable.

In conclusion, the work presented here provides a practical, scalable approach to the specific problem of learning a combined model for the effects of many compounds on many targets and demonstrates that the model can be combined with active machine learning methods to dramatically reduce the number of experiments needed to find compounds with desired target effects. Many variations on the approaches described here can be considered, including different predictive models, different feature sets and different active learning algorithms (such as information-theoretic scoring [27,28]). An exhaustive evaluation of these variations is beyond the scope of this paper, but we have firmly established that significant improvement in learning rates can be achieved. We believe active learning will be particularly important as drug development efforts increasingly consider variation among cell types and among individuals. The size of this experimental space clearly precludes exhaustive experimentation. The paradigm of exploring combinatorial experimental spaces through active learning is also widely applicable in biomedical research beyond drug discovery. This includes any study that seeks to determine the effects of large numbers of perturbations (such as genomic variation or exposure to compounds or inhibitory RNAs) on large numbers of molecular, cellular or histological behaviors (such as enzyme activities, cell shapes or motility, protein expression or localization). As the size of the experimental space grows, exhaustive experimentation becomes more impractical and active learning may be expected to provide even greater benefit.

## Methods

### Data preparation

Each assay from the PubChem database [29] contains gene target information, chemical identifier information and activity scores for all compounds tested in the assay. Various features describing the primary structure of the target protein were calculated using ProtParam [30], Protein Recon (http://reccr.chem.rpi.edu/Software/Protein-Recon/Protein-Recon-index.html) and Prosite [31]. In total, each assay was described by 388 features which described the target protein of that assay. All non-binary features were z-scored. The compounds in the assays were described with 1559 binary features calculated using OpenBabel [32] (http://openbabel.org). Assays from PubChem targeting human proteins with more than 15,000 compounds tested were manually annotated. For each assay, it was determined what type of effect was being detected for the target (inhibition, excitation, etc.) and the nature of the activity scores reported. The selected assays are found in Additional file 8. Only assays whose activity scores were scaled with a measured effect from the compound were kept for simulation. Activity scores were rescaled if necessary to a maximum of 100. For all assays testing for inhibition, scores were made negative. From the ~600,000 possible compounds, 20,000 were selected randomly for use in simulations of the active learning processes. The selected compounds are found in Additional file 9.

### Predictive model

#### Lasso regression

Linear regression models were trained with the following equations where ***Y***_*p*_ and ***X***_*p*_ are the vector of activity scores and matrix of compound features respectively from all executed experiments with protein *p*. The regression coefficients learned using lasso regression on the compound features to predict activity across target *p* are found in ***β***_*p*_. This method allows us to predict the effects from compounds on a single protein based on the features of that compound. Additionally, ***Y***_*d*_ and ***X***_*d*_ are the vector of activity scores and matrix of protein features respectively from all executed experiments with compound *d*. The regression coefficients learned using lasso regression on the protein target features to predict activity on all protein targets for compound *d* are found in ***β***_*d*_. These prediction methods are illustrated in Figure 1b-c.

(1) $$Y_{p}=X_{p}\beta_{p}$$

(2) $$Y_{d}=X_{d}\beta_{d}$$

Lasso selects a set of features that gives a fit where | ***β*** | < s. The penalty *s* was selected using cross validation for each linear regression model. Predictions made using only Equation 1 or 2 use *compound features only* (CFO) or *protein features only* (PFO) respectively. A combined prediction for the single compound tested in the single assay, *Y*_(*d,p*)_, was calculated by taking the mean of the predictions from Equations 1 and 2 which is illustrated in Figure 1d. We refer to this approach as the *combined compound-target (CCT) model*.

(3) $$Y_{\left(d,p\right)}=\left(Y_{d,p}+Y_{p,d}\right)/2$$

All regression models were trained using the Least Angle Regression method [33] implemented in SciKits (http://scikits.appspot.com). Penalties (*s*) were tested between 10^−4^ and 10^4^. Penalties were selected which minimized the mean squared error of five-fold cross validation within the training data for each model in each round of active learning.

### Selection methods

#### Greedy selection algorithm

Experiments were selected which had the greatest absolute value of predicted rank score (Y_(*d,p*)_). In some cases, no information was available to make a prediction for an experiment. If no prediction could be made from available data for an experiment, that experiment was predicted to have a rank score of zero. All experiments with equivalent predicted values were treated in random order.

#### Uncertainty sampling selection algorithm

For each assay, five CFO models were learned by subsampling the results available from observed experiments in that assay. For each unobserved experiment in an assay, a prediction is made using each of the five CFO models. Likewise, for each compound, five PFO models were learned by subsampling the results available from observed experiments for that compound. Across all unobserved assay in each compound a prediction was made using each PFO model. As a result, each unobserved experiment had five CFO predictions and five PFO predictions. Twenty-five predictions were calculated for each experiment by calculating the mean of each pair of CFO prediction and PFO prediction. If a model was impossible to calculate because there were no results from testing a compound *d* or no results from testing an assay *p*, only predictions from a single model were used. Experiments were selected which had the largest standard deviation of predictions because those were the experiments for which the model had the least confidence in prediction.

#### Density-based selection algorithm

Each experiment (target, compound) was represented by a single feature vector formed by concatenating the target features and the compound features for that experiment. For computational efficiency, a maximum of 2000 observed and 2000 unobserved experiments were used. Among the two thousand unobserved experiments, selections were made using a density-based sampling method [25] which attempted to choose experiments which were most distant (Euclidian distance) from already observed experiments and least distant from unobserved experiments. No predictions from a learned model were utilized for this selection method.

#### Diversity selection algorithm

Each experiment was represented by a single vector formed by concatenating the target features and the compound features for that experiment. A random set of 4000 experiments was clustered using the *k*-means algorithm (with *k* being the size of the batch desired, in our case 384). The experiment nearest to each centroid was selected for execution. No predictions from a learned model were utilized for this selection method.

#### Hybrid selection algorithms

For each round, half of the experiments were selected using one method and half were selected using another method.

#### Memory limited selection algorithms

When a predictive model was learned from observed data using Lasso regression, memory limitations were applied such that only experiments observed from the last *m* rounds of selection were used for training the predictive model.

### Availability of supporting data

The data supporting the results of this article are included within the article and its additional files.

## Abbreviations

CFO: Compound features only-model learned using information from only compound features; PFO: Protein features only-model learned using information from only target protein features; CCT: Combined compound-target model-model learned using information from both compound features and target protein features.

## Competing interests

Some of the technology described in this article is included under pending patent Application WO2012112534. The authors have financial interests in commercializing it.

## Authors’ contributions

JDK developed and implemented the initial approach, carried out the computational experiments, and wrote the initial draft of the manuscript. AWN contributed to the design of the experiments. RFM conceived the general design of the study, participated in the development of the approaches, and extensively edited the manuscript. All authors read and approved the final manuscript.

## Supplementary Material

Additional file 1 **Histogram of observed assay scores.** Scores range from −100 to 100 with negative scores indicating inhibitory effects and positive scores indicating activation effects. Scores of zero indicate no effect. Experiments with scores between −5 and 5 comprised 80% of the entire set. Less than 0.01% of scores were greater than 80 or less than −80. Click here for file

Additional file 2 **Descriptions of Target Protein Features and their Relevance.** The feature identifiers (Feature), feature source (Source) and feature descriptors (Description) are given in the spreadsheet. To determine the utility of features, a CCT model was trained for each of ten randomly selected subsets of 3% of all experimental results available. The result was that a regression model was trained for each compound (Figure 1c) which used target features to predict activity. For each feature, the proportion of regression models for which that feature was selected was calculated (SelectionFrequency) as well as its mean absolute coefficient (MeanAbsoluteBeta). Click here for file

Additional file 3 **Descriptions of Compound Features and their Relevance.** The feature identifiers (Feature), feature source (Source), feature descriptors (Content) and query string (PatternQuery) are given in the spreadsheet. To determine the utility of features, a CCT model was trained for each of ten randomly selected subsets of 3% of all experimental results available. The result was that a regression model was trained for each target (Figure 1b) which used compound features to predict activity. For each feature, the proportion of regression models for which that feature was selected was calculated (SelectionFrequency) as well as its mean absolute coefficient (MeanAbsoluteBeta). Click here for file

Additional file 4 **Evaluation of compound-target hit discovery for different active learning methods.** The average number of discoveries and standard error for 10 separate trials are shown. The methods were random choice (red), CCT with greedy selection (green), uncertainty sampling (blue), density-based sampling (cyan) and diversity selection (magenta). Click here for file

Additional file 5 **Evaluation of predictions for different active learning methods.** After each batch of experiments was chosen, a ROC curve was constructed by gradually raising the threshold on the predicted assay score at which an experiment was considered to be positive. The mean and standard error of the area under the ROC curve for prediction of positive experiments after each experiment is plotted for each regression method. The methods were random choice (red), CCT with greedy selection (green), uncertainty sampling (blue), density-based sampling (cyan) and diversity selection (magenta). Click here for file

Additional file 6 **Evaluation of compound-target hit discovery for different hybrid active learning methods.** The average number of discoveries and standard error for 10 separate trials are shown. The methods were random choice (red), CCT with greedy selection (green), hybrid greedy-uncertainty sampling (blue), hybrid greedy-density-based sampling (cyan) and hybrid greedy-diversity selection (magenta). Click here for file

Additional file 7 **Evaluation of compound-target hit discovery rates for compound libraries of different sizes.** Simulations were run using CCT with greedy selection for the exploration of 2% of the experimental space with subsets of the compounds of various sizes. These were repeated 10 times and the average and standard error for the percentage of discoveries made was calculated as a function of the percent of the experimental space sampled. The compound subsets were of the following sizes: 20,000 (black), 10,000 (dark gray) and 5,000 (light gray). Note that the rate of learning per fraction of experimental space is higher for larger compound libraries. Click here for file

Additional file 8 **List of PubChem Assays Utilized.** The PubChem assay ID (AssayID), data structure (DataStructure), target gene (GeneID), effect type (EffectType) and experiment type are listed for all assays used in this work. The structure of the data is shown to indicate how data were scaled. Some assays had activity scores which ranged from 0 – 100 and others needed to be rescaled. Some assay results were stored in a tiered format. For these assays, results from the lowest tier were used and rescaled to match a range from 0 – 100. Click here for file

Additional file 9 **List of PubChem Compounds Utilized.** This file contains a list of PubChem chemical IDs for all compounds used in this experiment. Click here for file
