# Supplementary figures and images for: Efficient discovery of responses of proteins to compounds using active learning
Source: BMC Bioinformatics. 2014 May 16;15:143. doi: 10.1186/1471-2105-15-143 (PMC4030446; doi:10.1186/1471-2105-15-143)

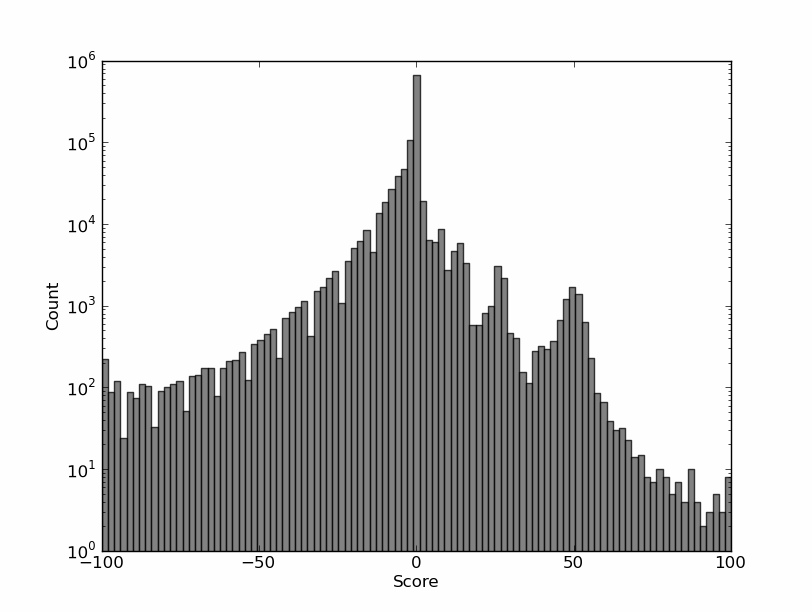

Supplement: Additional file 1 — Histogram of observed assay scores. Scores range from −100 to 100 with negative scores indicating inhibitory effects and positive scores indicating activation effects. Scores of zero indicate no effect. Experiments with scores between −5 and 5 comprised 80% of the entire set. Less than 0.01% of scores were greater than 80 or less than −80. [file 1471-2105-15-143-S1.png]

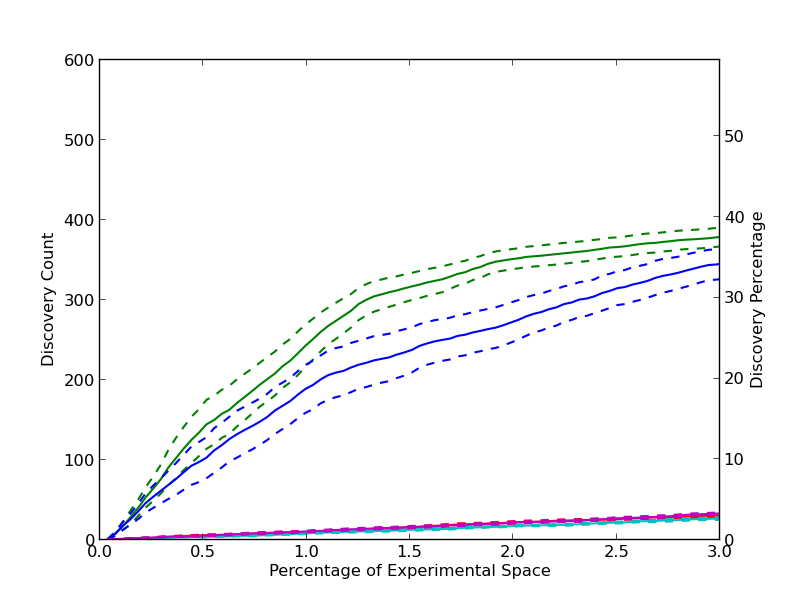

Supplement: Additional file 4 — Evaluation of compound-target hit discovery for different active learning methods. The average number of discoveries and standard error for 10 separate trials are shown. The methods were random choice (red), CCT with greedy selection (green), uncertainty sampling (blue), density-based sampling (cyan) and diversity selection (magenta). [file 1471-2105-15-143-S4.png]

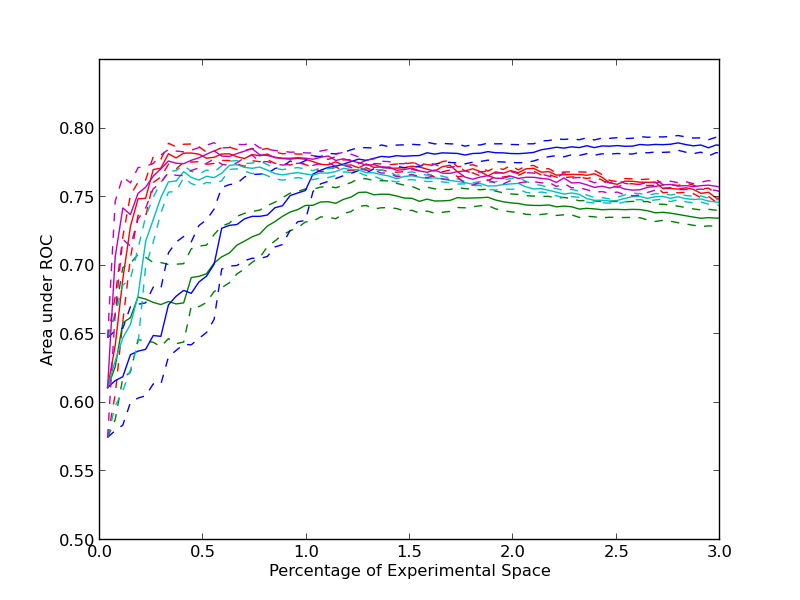

Supplement: Additional file 5 — Evaluation of predictions for different active learning methods. After each batch of experiments was chosen, a ROC curve was constructed by gradually raising the threshold on the predicted assay score at which an experiment was considered to be positive. The mean and standard error of the area under the ROC curve for prediction of positive experiments after each experiment is plotted for each regression method. The methods were random choice (red), CCT with greedy selection (green), uncertainty sampling (blue), density-based sampling (cyan) and diversity selection (magenta). [file 1471-2105-15-143-S5.png]

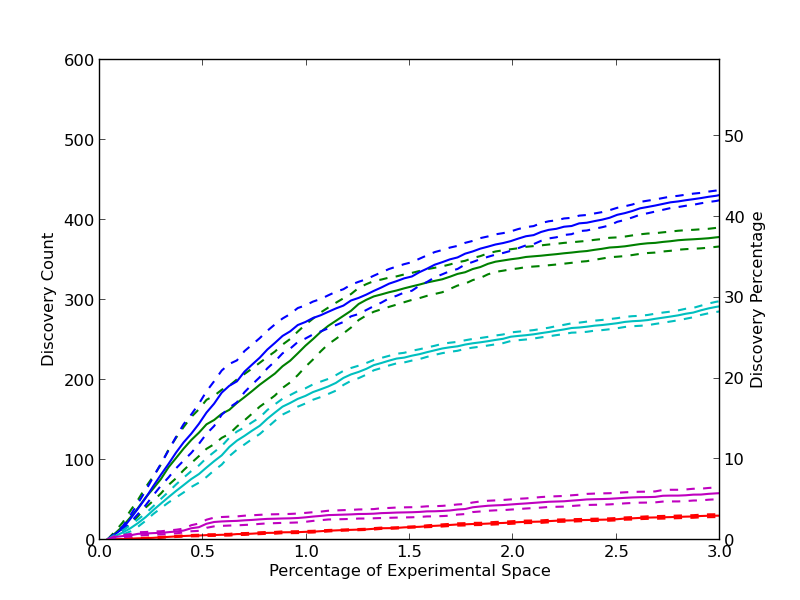

Supplement: Additional file 6 — Evaluation of compound-target hit discovery for different hybrid active learning methods. The average number of discoveries and standard error for 10 separate trials are shown. The methods were random choice (red), CCT with greedy selection (green), hybrid greedy-uncertainty sampling (blue), hybrid greedy-density-based sampling (cyan) and hybrid greedy-diversity selection (magenta). [file 1471-2105-15-143-S6.png]

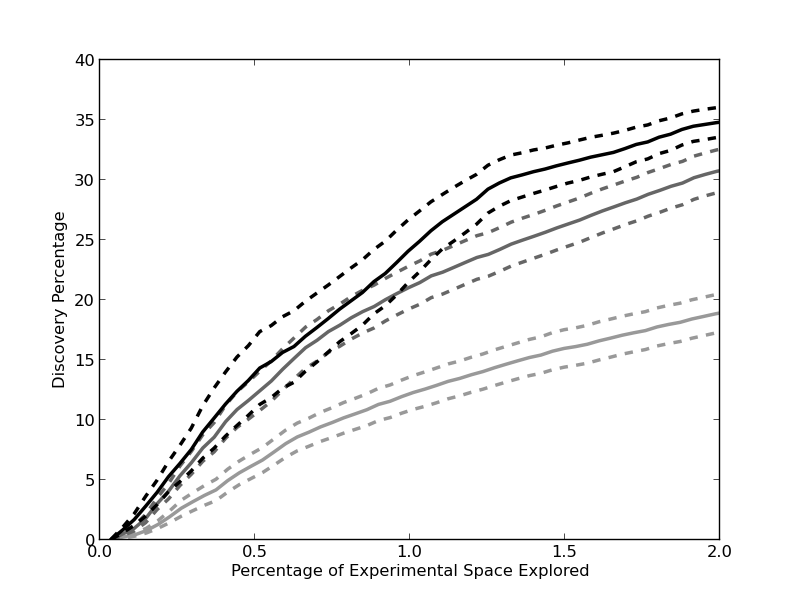

Supplement: Additional file 7 — Evaluation of compound-target hit discovery rates for compound libraries of different sizes. Simulations were run using CCT with greedy selection for the exploration of 2% of the experimental space with subsets of the compounds of various sizes. These were repeated 10 times and the average and standard error for the percentage of discoveries made was calculated as a function of the percent of the experimental space sampled. The compound subsets were of the following sizes: 20,000 (black), 10,000 (dark gray) and 5,000 (light gray). Note that the rate of learning per fraction of experimental space is higher for larger compound libraries. [file 1471-2105-15-143-S7.png]
